# Study on Quenching Characteristics and Resistance Equivalent Estimation Method of Second-Generation High Temperature Superconducting Tape under Different Overcurrent

**DOI:** 10.3390/ma12152374

**Published:** 2019-07-25

**Authors:** Siyuan Liang, Li Ren, Tao Ma, Ying Xu, Yuejin Tang, Xiangyu Tan, Zheng Li, Guilun Chen, Sinian Yan, Zhiwei Cao, Jing Shi, Leishi Xiao, Meng Song

**Affiliations:** 1 State Key Laboratory of Advanced Electromagnetic Engineering and Technology, School of Electrical and Electronic Engineering, Huazhong University of Science and Technology, Wuhan 430074, China; 2 School of Electrical Engineering, Beijing Jiaotong University, Beijing 100044, China; 3 Electric Power Research Institute of Guangdong Power Grid Corporation, Guangzhou 510080, China

**Keywords:** superconducting tape, quench, R-SFCL, AC and DC overcurrent, experiment, finite element method (FEM), numerical modeling

## Abstract

In this paper, through AC and DC overcurrent tests on second generation high temperature superconducting tape (2G HTS tape), we respectively summarize the typical types of quenching resistance and corresponding quenching degree, in which there are three types under AC overcurrent and two types under DC overcurrent. According to experimental results, a rule was found that, when 2G HTS tape quenches to normal state, the relationship between quenching resistance and joule heat generated from 2G HTS tape presents a fixed trend line, and the influence of liquid nitrogen can be ignored. Then, the characteristics and rules of quenching resistance found in experiments are well explained and confirmed by a detailed 3D finite element model of 2G HTS tape including electromagnetic field and thermal field. Finally, based on above works, our group proposes a new equivalent method to estimate the quenching resistance, where the results of AC and DC overcurrent experiments can be equivalent to each other within a certain range. Compared with FEM, the method has the following advantages: (i) The method is simple and easy to implement. (ii) This method combines precision and computational efficiency. (iii) With superconducting tape quenching to normal state, this method presents a good consistency with experimental results.

## 1. Introduction

With the discovery of the cuprate-based high temperature superconductors, first generation high temperature superconducting (1G HTS) tapes represented by BSCCO (Bismuth Strontium Calcium Copper Oxide) Ag-sheathed conductors and second-generation high temperature superconducting (2G HTS) tapes represented by YBCO (Yttrium Barium Copper Oxide) coated conductors have appeared successively [1,2]. Compared with 1G HTS tape, through the improvement of manufacturing process, YBCO HTS tapes have the advantages of higher current density, lower alternating current (AC) loss and lower theoretical cost [3,4,5,6,7], which provide an opportunity for the high power application of superconducting devices applied in the field of electric power.

When current exceeds critical current, superconducting tape will automatically quench and switch to resistance state, which can limit short circuit fault current in power system. Based on the quenching characteristics of YBCO material, resistance type superconducting fault current limiter (R-SFCL) has advantages of simple structure, automatic trigger and fast response. Therefore, R-SFCL can be widely applied to limit short circuit fault current in multiple application scenarios, such as ship power grid [8], railway direct current (DC) traction systems [9], microgrid system [10,11], DC power grid [12,13,14] and so on.

However, HTS tape is very sensitive to magnetic field, temperature field and stress field. The complex working environment formed by multiple physical fields has a great influence on the homogeneity and stability of superconducting tape. Therefore, the applications of R-SFCL in power system requires the manufacture of superconducting tape with excellent performance and obvious quenching characteristics. It is necessary to focus on studying the quenching characteristic of YBCO material to realize the engineering application of R-SFCL. At present, many scholars have conducted extensive and in-depth research on the different characteristics of YBCO superconducting tape including quenching behavior under AC or DC overcurrent [15,16,17,18,19,20,21,22], quench propagation [23,24,25,26], quenching recovery [27,28,29,30], thermal and mechanical properties [31,32], minimum quench energy [33,34], maximum operating condition [35], and the effect of different materials on quenching behavior [36,37,38], which provide a solid foundation for the use of superconducting tape in design and fabrication of R-SFCL. However, there are few studies on common characteristics of quenching resistance of 2G HTS tape under AC and DC overcurrent, which is necessary and basic for R-SFCL design and research.

In addition, it is very difficult to carry out quenching test on a large-scale DC R-SFCL prototype in practical engineering applications before installed in large capacity voltage source converter (VSC) or modular multilevel converter (MMC) high voltage direct current (HVDC) projects. The transmission power of the HVDC projects usually reach hundreds of megawatts. It is very risky to carry out short circuit fault for testing R-SFCL in the existing DC system projects. Meanwhile, the economic and technical cost of manufacturing large-capacity DC test platform for R-SFCL is very high. On the contrary, the technology of large-scale AC experiment platform is mature and its cost is lower. At present, with the support of national key research and development plan, a large-scale DC R-SFCL is being developed in China, which is planned to be installed in Nanao’s ±160 kV MMC three-terminal HVDC system. If the common characteristics and internal relations of 2G HTS tape under different type overcurrent is found, the AC overcurrent experimental results of the R-SFCL can be used to evaluate quenching resistance of R-SFCL under DC overcurrent.

In this study, a kind of YBCO tape produced by Shanghai Superconductor Company for R-SFCL was tested under AC and DC overcurrent. By comparing the experimental results of AC and DC overcurrent test, the common characteristics and laws of quenching resistance variation were found under different overcurrent in 77 K liquid nitrogen immersion environment. The same conclusions were obtained through the simulation of YBCO three-dimensional (3D) finite element model. Based on the findings, a new estimation method is proposed, which can accurately reflect and estimate the quenching resistance within a certain range, whose feasibility and availability was verified by comparing with experimental results. Furthermore, it provides theoretical basis and experimental support for AC equivalent test method before a large-scale DC R-SFCL connected to DC power grid.

## 2. 2G HTS Tape

The YBCO superconducting tapes used in the experiment are produced by Shanghai Superconductor Company (Shanghai, China) [39], which are mainly adopted in R-SFCL. In general, the typical structure of YBCO tape consists of copper stabilizer, silver stabilizer, YBCO layer and Hastelloy substrate. In addition, a buffer layer exists between YBCO layer and Hastelloy substrate.

**Figure 1:**
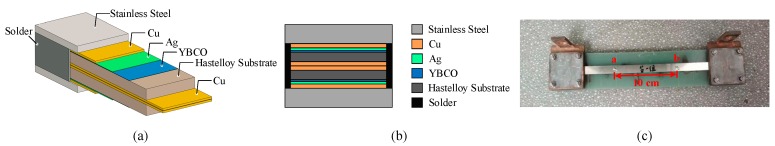
A 2G HTS tape produced by Shanghai Superconductor Company: (**a**) 3D structural diagram; (**b**) sectional view; and (**c**) the sample prepared for experiment.

The 2G HTS tape is 12 mm wide with a two-core configuration, including two typical structures of YBCO tape with copper stabilizer on both side, which are stacked back-to-back and encapsulated with stainless steel layer, as shown in Figure 1a,b (buffer layer is omitted). This structure can increase self-field critical current, which is suitable for power systems with high current. The corresponding basic parameters of the 2G HTS are listed in Table 1. As shown in Figure 1c, the middle 10 cm area of tape samples are selected for voltage measurement in AC and DC overcurrent tests.

## 3. AC and DC Overcurrent Experiment

**Figure 2:**
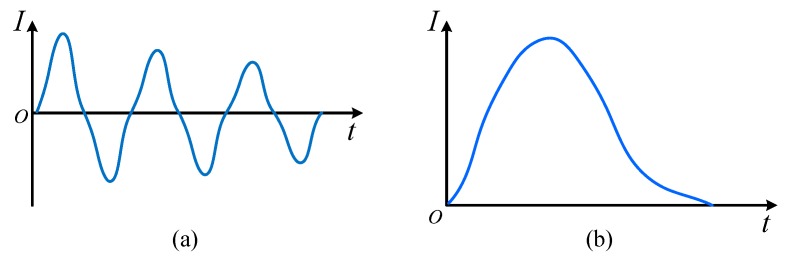
Two type basic waveforms in the experiment: (**a**) AC overcurrent; and (**b**) DC overcurrent.

In a strict sense, a current whose magnitude and direction change periodically with time is defined as AC current, while a current whose magnitude and direction remain constant is defined as a DC current. However, in the actual system short circuit fault, the overcurrent will present irregular, even drastic changes. Therefore, in this paper, we define AC and DC overcurrent in a broad sense. The overcurrent in constant direction is DC overcurrent, and the overcurrent in direction varying with time is AC overcurrent. The two kinds of overcurrent analyzed in the experiments are shown in Figure 2.

### 3.1. AC and DC Overcurrent Experiment Platforms

**Figure 3:**
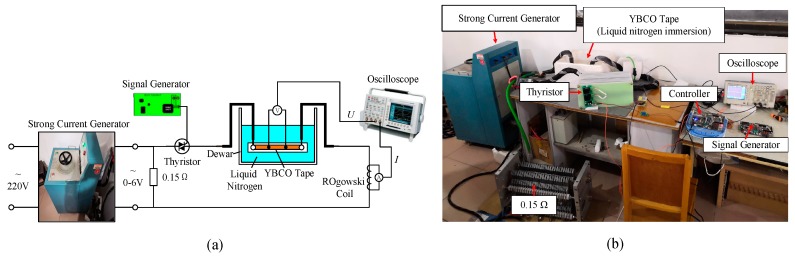
AC overcurrent test platform: (**a**) circuit diagram of test platform; and (**b**) physical map of test platform.

**Figure 4:**
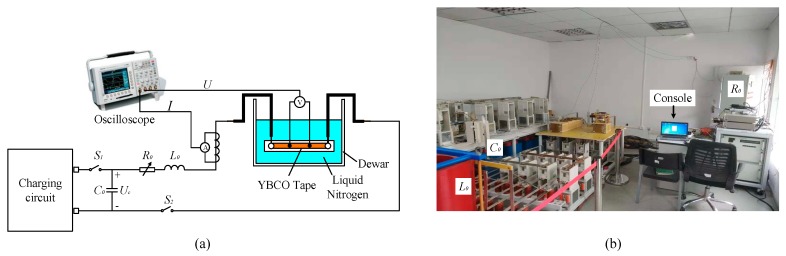
DC overcurrent test platform: (**a**) circuit diagram of test platform; and (**b**) physical map of test platform.

The AC and DC overcurrent test platforms were, respectively, built for studying quenching characteristics of YBCO tape, as shown in Figure 3 and Figure 4, and their basic corresponding parameters are listed in Table 2. The parameters setting of AC and DC overcurrent experiments are listed in Table 3. The YBCO tape to be tested was immersed in liquid nitrogen and cooled to a superconducting state at 77 K.

The AC overcurrent test platform was equipped with a 20 kVA strong current generator, which can turn the 220 V power supply into a 6 V high-power current source with a maximum output of 3 kA. Output capacity was adjusted by strong current generator. The on–off state of thyristor decides duration of AC overcurrent, which was controlled by the signal generator and controller.

The DC overcurrent test platform was the series circuit of resistance *R*, inductance *L* and capacitance *C* (RLC). The RLC series circuit was used to approximately equivalent and simulate the capacitor discharging process of VSC and MMC systems in the DC side short-circuit fault [40,41]. When S_1_ is in the closed state and S_2_ is in the disconnected state, charging circuit begins to charge the capacitor until the voltage *U_C_* reaches a set value. When S_1_ is disconnected and S_2_ is closed, *R*_0_, *L*_0_ and *C*_0_ form a closed loop and generate a DC overcurrent for the test of YBCO tape. Peak value, rising slope and duration of DC overcurrent waveform vary with adjusting *U_C_*, *L*_0_ and *R*_0_.

### 3.2. The results and Analysis of AC Overcurrent Experiment

**Figure 5:**
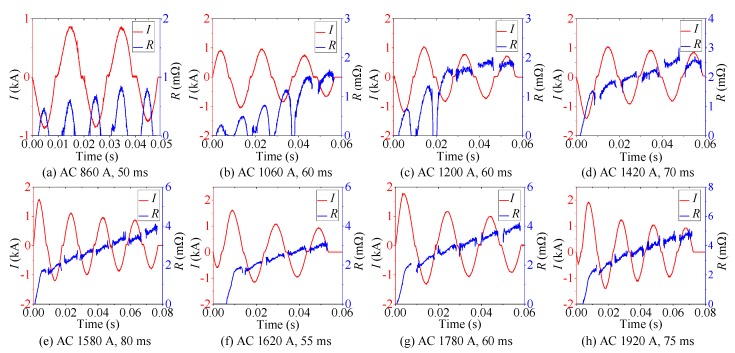
The corresponding experimental results of AC overcurrent test with different amplitude of AC overcurrent.

Eight groups of AC overcurrent tests on YBCO tape were carried out by adjusting the amplitude and duration of overcurrent. The amplitude of overcurrent gradually increased from 860 A to 1920 A. The range of overcurrent duration was 50–80 ms. The eight-group experimental results are shown in Figure 5. The AC experimental analysis is summarized in Table 4.

As shown in Figure 5a, continuous half-wave type quenching resistance suggests that the quenching state of superconducting tape cannot be maintained, which is unstable and discontinuous. With AC overcurrent decreasing, the superconducting tape returns back to superconducting state and quenching resistance tends to 0 Ω. Therefore, this quenching state is inadequate. At this time, the state of superconducting tape is defined as partial resistive state.

As shown in Figure 5b,c, there are two types resistance waveform under this AC overcurrent level, continuous half-wave type and incremental curve type. In initial stage, superconducting tape recovers rapidly after quenching. Then, superconducting tape begins to quench continuously and transits from fluctuation to continuous increment. It indicates that the quenching degree of superconducting tape gradually deepens and further transits to normal state. This quenching stage of superconducting tape is defined as transition stage, where the state of superconducting tape starts to transit from partial resistive state to normal state.

As shown in Figure 5d–h, incremental curve type quenching resistance reflects that superconducting tape responds to quench continuously without fast recovering under this AC overcurrent level. The quenching state in superconducting tape is adequate and stable, which is considered as complete quenching. It indicates that the superconducting tape is in normal state.

### 3.3. The Results and Analysis of DC Overcurrent Experiment

**Figure 6:**
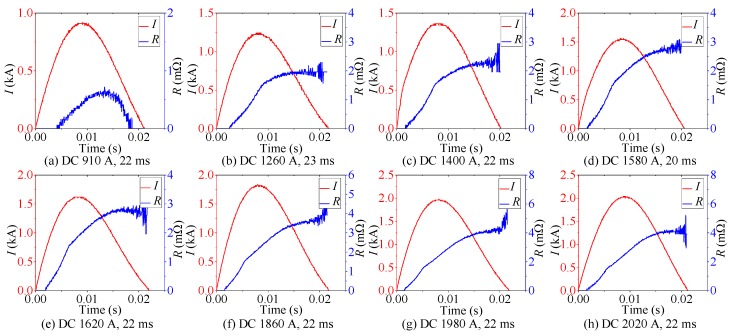
The corresponding experimental results DC overcurrent test with different amplitude of DC overcurrent.

The eight-group DC experimental results are presented in Figure 6. The amplitude of overcurrent gradually increases from 910 A to 2020 A. The range of overcurrent duration is 20–23 ms. The DC experimental analysis is summarized in Table 5.

As shown in Figure 6a, half-wave type quenching resistance suggests that the quenching state of superconducting tape cannot always be maintained under this DC overcurrent level. When DC overcurrent decreases, superconducting tape returns to superconducting state rapidly and quenching resistance tends to 0 Ω. Therefore, this quenching state is inadequate, which is considered as incomplete quenching. Similar to the result of AC experiment in Figure 5a, the superconducting tape is in partial resistive state.

As shown in Figure 6b–h, the quenching degree of superconducting tape is strengthened with the peak value *I_Pmax_* of DC overcurrent increasing. Because DC overcurrent has no zero crossing before current declines to 0, quenching resistance is incremental curve type. They reflects that superconducting tape quenches continuously without fast recovering under this DC overcurrent level. During this stage, the superconducting tape completely quenches to normal state and presents normal resistance.

### 3.4. Comparative Analysis of Quenching Resistance under AC and DC Overcurrent

**Figure 7:**
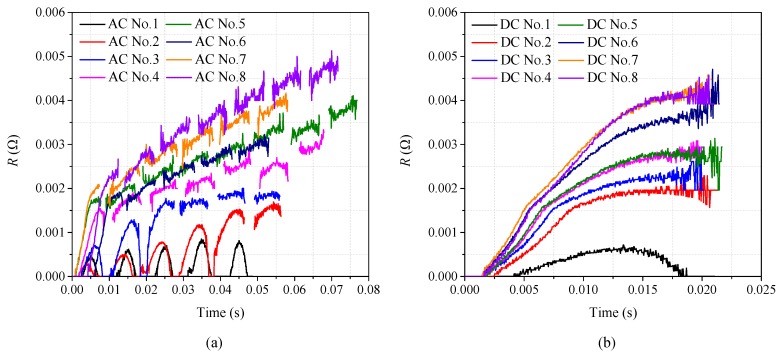
The *R-t* curves of superconducting tape under different overcurrent: (**a**) the *R-t* curves under AC overcurrent; and (**b**) the *R-t* curves under DC overcurrent.

According to the above results of AC and DC overcurrent experiment shown in Figure 5 and Figure 6, the corresponding *R-t* curves of superconducting tape are obtained, respectively, shown in Figure 7a,b. The *R-t* curves reflect resistance varying with time under different overcurrent. With the increase of overcurrent amplitude, the quenching degree of superconducting tape is enhanced. However, the rising slope and steady-state value of *R-t* curves vary with waveform type, amplitude and duration of impact current. Therefore, *R-t* relationship cannot be used to describe the variation of quenching resistance in superconducting tape.

**Figure 8:**
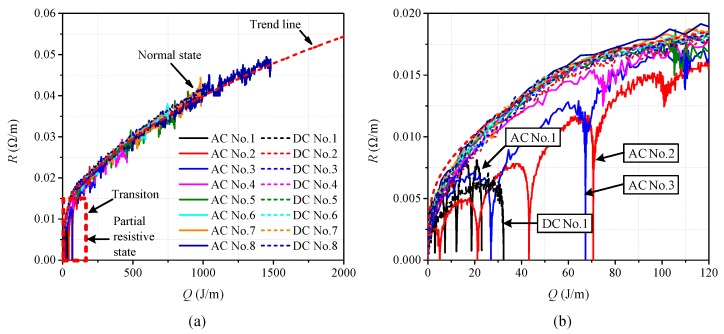
The *R-Q* curves of superconducting tape under AC and DC overcurrent: (**a**) the comparison diagram of *R-Q*; and (**b**) partial enlarged drawing of the *R-Q* curves.

Temperature plays a key role in quenching resistance and quenching degree in superconducting tape. In a certain cryogenic medium, joule heat accumulation is the fundamental cause of temperature rise. Without considering the variation of cryogenic medium, the main factor affecting the temperature of superconducting tape should be the joule heat generated during the quenching process, which is the result of comprehensive effect from quenching resistance, current and quenching duration. The generated joule heat *Q* of the superconducting tape varying with time can be calculated by Equation (1), whose unit is joule (J).

(1) $$Q\left(t_{2}\right)=\int_{t_{1}}^{t_{2}}I^{2}R\left(t\right)\;dt$$

where *t*_1_ is the initial time of quenching, *R*(*t*) is quenching resistance corresponding to time, and the range of time *t* is [*t*_1_, *t*_2_]. According to sixteen-group data of experimental results, *R-Q* curves of per meter superconducting tape are obtained, where dependent variable *R* is the quenching resistance of per meter superconducting tape and independent variable *Q* is the generated joule heat of per meter superconducting tape, as shown in Figure 8.

Figure 8a is the comparison diagram of *R-Q* curves with different overcurrent, and Figure 8b is the partial enlarged drawing of Figure 8a. It is quite clear that twelve *R-Q* curves obtained are basically overlapping and their trend line are consistent under AC and DC overcurrent except the results of DC No. 1, AC No. 1, AC No. 2 and AC No. 3. According to the classification in Section 3.2 and Section 3.3, DC No. 1 and AC No. 1 belong to partial resistive stage of superconducting tape, while AC No. 2 and No. 3 belong to transition stage of superconducting tape.

Therefore, the experimental results present a common trait. When YBCO superconducting tape completely quenches in a cryogenic medium, though superconducting tape is subjected to different overcurrent, the quenching resistance *R* has an intrinsic fixed relationship with the generated joule heat *Q* in superconducting tape. In other words, *Q* is the deciding factor of quenching resistance, while overcurrent is the only inducing factor of quenching resistance. The preliminary finding suggests that, during the complete quenching stage of superconducting tape, quenching resistance can be described by a fixed *R-Q* curve, which can be obtained from experiments in comprehensive consideration of current, resistance and duration. Therefore, *R* should satisfy an incremental relationship with *Q* when superconducting tape tends to complete quenching, described by Equation (2), which also can be reflected by an *R-Q* curve.

*R* ∝ *Q*(*I*, *R*, *t*) (2)

Based on experimental phenomena of superconducting tapes, the quenching resistance characteristics and laws of superconducting tapes are summarized. However, experimental data alone are insufficient to provide adequate physical explanations for the phenomena and laws. Therefore, as presented in Section 4, commercial finite element software COMSOL Multiphysics was adopted to establish 3D simulation model of 2G HTS tape for exploring the criteria of quenching resistance classification, validating the *R-Q* curve and providing corresponding physical explanations.

## 4. Simulation Study on 2G HTS Tape Quenching under AC and DC Overcurrent

### 4.1. 3D Finite Element Model of 2G HTS Tape

**Figure 9:**
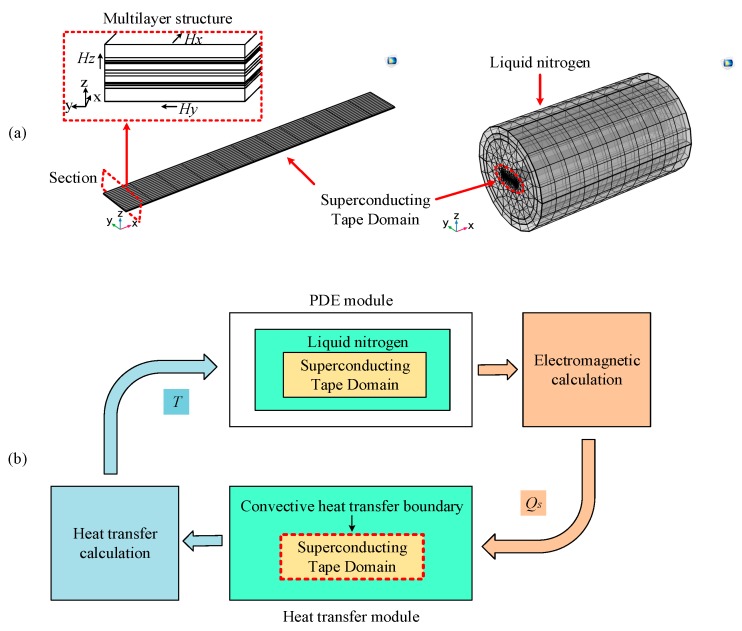
The basic structure of the simulation model: (**a**) geometric structure of superconducting tape; and (**b**) the coupling structure of electromagnetic thermal.

In software COMSOL Multiphysics, according to the basic parameters of the superconducting tape listed in Table 1, the 3D multilayer structure of the 2G HTS tape was established as superconducting tape domain, whose surface is covered with liquid nitrogen domain. A combination of custom partial differential equation module (PDE module) and heat transfer module were adopted to perform transient coupling calculation of electric field, magnetic field and thermal field. PDE module was used to carry out electromagnetic calculation. Heat transfer module was used to carry out thermal calculation. The two modules were coupled by joule heat and temperature. The basic structure of the simulation model is shown in Figure 9.

### 4.1.1. 3D Electromagnetic Model

The 3D electromagnetic calculation was based on Maxwell equations and H equation [42] with good convergence. The Faraday’s law and the ampere-law equation, respectively, are described as Equations (3) and (4).

(3) $$\nabla \times \vec{E}=-\frac{\partial \vec{B}}{\partial t}=-\mu_{0}\mu_{r}\frac{\partial \vec{H}}{\partial t}$$

(4) $$\nabla \times \vec{H}=\vec{J}+\frac{\partial \vec{D}}{\partial t}$$

where $\vec{E}$ is electric field intensity, $\vec{B}$ and $\vec{H}$, respectively, are magnetic induction intensity and magnetic field intensity, and $\vec{J}$ and $\vec{D}$ are conduction current density and displacement current density. Since the conduction current density is much higher than displacement current density in the superconducting tape, the differential term of displacement current with respect to time in Equation (4) can be ignored, thus $\nabla \times \vec{H}=\vec{J}$.

Because $B\;=\mu_{0}\mu_{r}H$ and E=rJ, the partial differential Equation (5) can be obtained according to Equations (3) and (4), where *μ*_0_ is permeability of vacuum, 4π × 10^−7^ H/m, *μ_r_* is relative permeability, and *r* is electric resistivity.

(5) $$\nabla \times \left(r\cdot \nabla \times \vec{H}\right)=-\mu_{0}\mu_{r}\frac{\partial \vec{H}}{\partial t}$$

where $\nabla \times \vec{H}=J_{x}\vec{e_{x}}+J_{y}\vec{e_{y}}+J_{z}\vec{e_{z}}$. *J_x_*, *J_y_* and *J_z_* are the components of *J* in the x, y, and z directions, which can be expressed by Equations (6).

(6) $$J_{x}=\frac{\partial H_{z}}{\partial y}-\frac{\partial H_{y}}{\partial z},\;J_{y}=\frac{\partial H_{x}}{\partial z}-\frac{\partial H_{z}}{\partial x},\;J_{z}=\frac{\partial H_{y}}{\partial x}-\frac{\partial H_{x}}{\partial y}$$

Combining with Equations (5) and (6), the governing equation of electromagnetic calculation are obtained as follows.

(7) $$\nabla \cdot \left[\begin{array}{ccc} 0 & -E_{z} & E_{y}\\ E_{z} & 0 & -E_{x}\\ -E_{y} & E_{x} & 0 \end{array}\right]\left[\begin{array}{l} \vec{e_{x}}\\ \vec{e_{y}}\\ \vec{e_{z}} \end{array}\right]+\frac{\partial }{\partial t}\left[\begin{array}{ccc} \vec{e_{x}} & \vec{e_{y}} & \vec{e_{z}} \end{array}\right]\left[\begin{array}{ccc} \mu_{0}\mu_{r} & 0 & 0\\ 0 & \mu_{0}\mu_{r} & 0\\ 0 & 0 & \mu_{0}\mu_{r} \end{array}\right]\left[\begin{array}{l} H_{x}\\ H_{y}\\ H_{z} \end{array}\right]=0$$

where $\nabla =[\frac{\partial }{\partial x},\;\frac{\partial }{\partial y},\;\frac{\partial }{\partial z}]$. $\vec{e_{x}}$, $\vec{e_{y}}$, and $\vec{e_{z}}$ are, respectively, the unit direction vector of *x*, *y*, and *z* directions. *E_x_*, *E_y_*, and *E_z_* are the components of *E* in the *x*, *y*, and *z* directions. *H_x_*, *H_y_*, and *H_z_* are the components of *H* in the x, y, and z directions, which are set as dependent variables in PDE module.

Overcurrent was used as model excitation in simulation. As a composite conductor, each layer of the superconducting tape was assumed to be parallel structure. Therefore, the current constraint condition of the model should meet Equation (8), which is load on the cross section of superconducting tape by pointwise constraint.

(8) $$I_{input}=I_{SC}+I_{Cu}+I_{Ag}+I_{Sub}+I_{St}$$

where *I_input_* is total current excitation, *I_SC_* is the current in YBCO layer, *I_Cu_* is the current in copper layer, *I_Ag_* is the current in silver layer, *I_Sub_* is the current in Hastelloy substrate, and *I_St_* is the current in stainless steel layer.

In the quenching model of superconducting tape, the calculation of YBCO resistivity is crucial; a piecewise formula for YBCO resistivity was adopted, which is expressed as Equations (9). Critical temperature *T_c_* is used as criterion for state transition. When *T* ≤ *T_c_*, the superconducting tape is considered as incomplete quenching, whose YBCO resistivity adopts the classical method of parallel equivalent electrical circuit [43]. When *T* > *T_c_*, the superconducting tape is considered as complete quenching, whose YBCO resistivity adopts constant resistivity *r_norm_*.

(9) $$r_{YBCO}=\left\{ \begin{array}{cc} \frac{\left(r_{1}+r_{2}+r_{0}\right)r_{norm}}{r_{0}+r_{1}+r_{2}+r_{norm}} & \;T\leq T_{c}\\ r_{norm} & \;T>T_{c} \end{array}\right.$$

where *r*_0_ is residual resistivity [44], *r*_1_ is expressed as Equation (10), *r*_2_ is expressed as Equation (11). According to the authors of [42], *n*_1_, *n*_2_ and *E*_0_ are fitting parameters of two power-law relations, and *k* can be calculated based on *r*_1_(3*J_c_*) = *r*_2_(3*J_c_*).

(10) $$r_{1}=\left\{ \begin{array}{ll} 0 & \left|J\right|<J_{c}\\ \frac{E_{0}}{\left|J\right|}{\left(\frac{\left|J\right|}{J_{c}}-k\right)}^{n_{1}} & \left|J\right|\geq J_{c} \end{array}\right.$$

(11) $$r_{2}=\left\{ \begin{array}{ll} 0 & \left|J\right|<kJ_{c}\\ \frac{E_{0}}{\left|J\right|}{\left(\frac{\left|J\right|}{J_{c}}-k\right)}^{n_{2}} & \left|J\right|\geq kJ_{c} \end{array}\right.$$

According to the authors of [45], when the temperature *T* of YBCO layer is below critical temperature *T_c_*, the critical current density *J_c_* of YBCO material is affected by temperature *T*. When *T* is above *T_c_*, *J_c_* is considered as 0 A/m^2^. The relationship can be expressed by the improved typical Equation (12) in [43].

(12) $$J_{c}\left(T\right)=\left\{ \begin{array}{cc} J_{c0}{\left(\frac{T_{c}-T}{T_{c}-T_{ref}}\right)}^{\alpha } & \left(T_{ref}<T<T_{c}\right)\\ 0 & \left(T_{c}\leq T\right) \end{array}\right.$$

where *T_ref_* is initial ambient temperature or cryogenic medium temperature of the superconducting tape. *J_c_*_0_ is critical current density corresponding to *T_ref_*. In this simulation, superconducting tapes were cooled by liquid nitrogen immersion. Therefore, *T_ref_* was set to 77 K. The relevant simulation parameters of YBCO resistivity are listed in the Table 6.

#### 4.1.2. Heat transfer Model

In heat transfer calculation, because superconducting tape was immersed in liquid nitrogen, which is without obvious flowing, a heat balance Equations (13) in the module of Heat Transfer in Solids in COMSOL Multiphysics was used to calculate the thermal characteristics of the entire superconducting tape region including YBCO layer, Cu layer, Ag layer, stainless steel and Hastelloy substrate.

(13) $$\left\{ \begin{array}{l} q_{s}=\rho c\frac{\partial T}{\partial t}+\nabla \cdot q\\ q_{s}=E_{x}J_{x}+E_{y}J_{y}+E_{z}J_{z}\\ q=-k\nabla T \end{array}\right.$$

where *q_s_* is volume power density, with unit W/m^3^; *ρ* is mass density, with unit kg/m^3^; *c* is specific heat capacity, with unit J/(kg·K); *q* is conduction heat flux, with unit W/m^2^; and *k* is heat transfer coefficient, with unit W/(m^2^·K).

**Figure 10:**
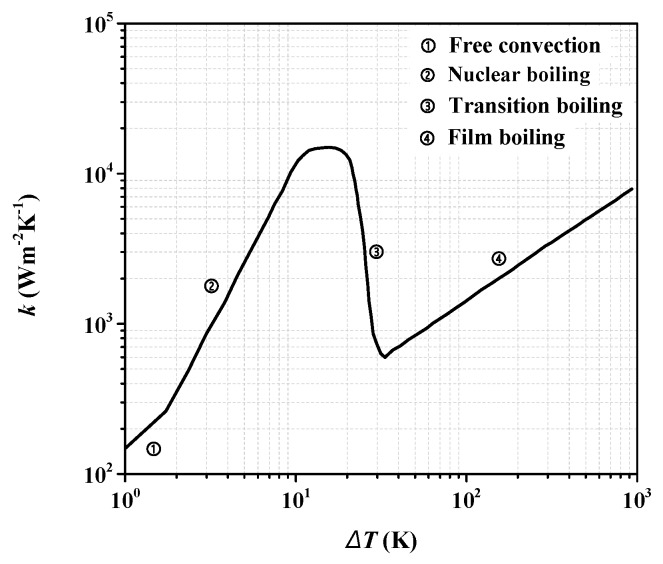
Heat transfer coefficient curve of liquid nitrogen.

In the simulation, the initial temperature of thermal solution domain was set to 77 K, and the boundary condition of the superconducting tape surface was set to Heat Flux. To simulate the heat exchange process between superconducting tape surface and liquid nitrogen, a heat transfer coefficient curve in Heat Flux was set on the interface between equivalent superconducting tape and liquid nitrogen [43,46,47], which is a common method to simulate heat exchange process between liquid nitrogen and superconducting tape surface at different temperatures. As shown in Figure 10, this is a heat transfer coefficient curve of liquid nitrogen including free convection, nuclear boiling, transition boiling and film boiling [48].

### 4.2. Verification of the 3D Finite Element Model

**Figure 11:**
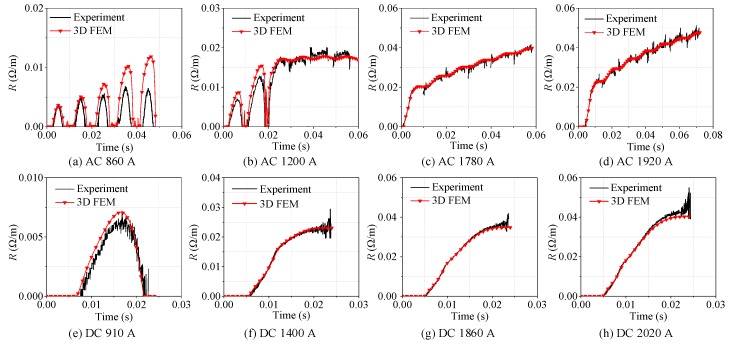
Comparison of experimental and simulation results.

As shown in Figure 11a–d, four-group quenching data of superconducting tape under different AC overcurrent were selected for comparison between experiment and simulation, covering three types quenching resistance waveforms of superconducting tapes under AC overcurrent. Figure 11e–h compares the results of experiment and simulation, covering two types quenching resistance waveforms of superconducting tape under DC overcurrent.

Through waveform comparison between experiment and simulation, it can be found that the simulation phenomena are basically consistent with the classification of experimental results, where the model can correctly describe the different types of quenching resistance under different overcurrent. Therefore, the 3D finite element model can correctly reflect the quenching state of the superconducting tape, and contributes to research the internal evolution process of superconducting tape under AC and DC overcurrent.

### 4.3. Thermal Characteristics and Current Distribution Characteristics of 2G HTS Tape Quenching

#### 4.3.1. Resistance, Thermal and Current Distribution Characteristics under AC Overcurrent

To analyze the transient characteristics of superconducting tape under AC overcurrent and obtain corresponding common laws, it was necessary to select and analyze the three typical quenching resistance waveforms of superconducting tape found in Section 3.2 under AC overcurrent, including half-wave type, half-wave and half-incremental curve type and incremental curve type. Therefore, the simulation results with AC 860 A, AC 1200 A and AC 1780 A were selected.

**Figure 12:**
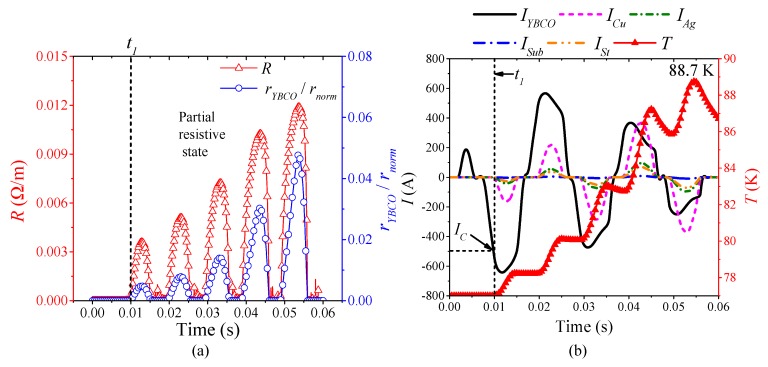
The simulation analysis with AC 860 A overcurrent: (**a**) the quenching resistance characteristics; and (**b**) the thermal characteristics and current distribution characteristics.

Figure 12 is the simulation results of the superconducting tape quenching under AC 860 A, which represent the characteristics of continuous half-wave type quenching resistance in partial resistive state. According to Figure 12a, when 0 ≤ *t* < *t*_1_, the superconducting tape is in superconducting state with 0 Ω, and current only flows through YBCO layer. When *t*_1_ ≤ *t*, current begins to exceed the critical current *I_C_* which results in YBCO layer quenching. However, the ratio of real-time resistivity to normal resistivity of YBCO is very low, no more than 0.07. It indicates that the superconducting tape is in partial resistive state. As shown in Figure 12b, during *t*_1_ to 60 ms, the temperature of YBCO layer rises continuously in fluctuation, but the maximum value of temperature is only 88.7 K, which deso not exceed the critical temperature *T_C_* of YBCO layer. Therefore, during the whole quenching process, the quenching resistivity of YBCO layer is in the first stage in Equation (9), which causes the current to be diverted to other layers. The resistivity of YBCO layer fluctuates with the rise and fall of AC overcurrent, which results in the superconducting tape producing the corresponding continuous half-wave type quenching resistance. During initial quenching, the current distribution among material layers is: *I_YBCO_* > *I_Cu_* > *I_Ag_* > *I_St_* > *I_Sub_*. With the temperature of YBCO layer rising, the quenching resistivity of YBCO layer increases, and the ordering of current in proportion is: *I_Cu_* > *I_YBCO_* > *I_Ag_* > *I_St_* > *I_Sub_*. 

**Figure 13:**
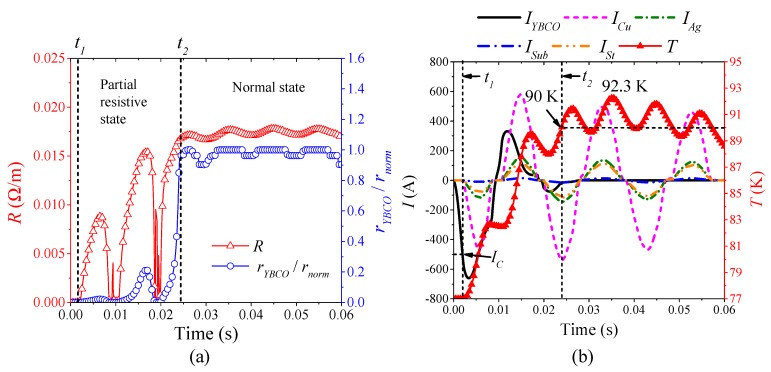
The simulation analysis with AC 1200 A overcurrent: (**a**) the quenching resistance characteristics; and (**b**) the thermal characteristics and current distribution characteristics.

Figure 13 is the simulation results of the superconducting tape quenching under AC 1200 A, which represents the characteristics of continuous half-wave and half-incremental curve type quenching resistance in transition state. According to Figure 13a, when 0 ≤ *t* < *t*_1_, the superconducting tape is in superconducting state with 0 Ω, current only flow through YBCO layer. When *t*_1_ ≤ *t* < *t*_2_, the ratio of real-time resistivity to normal resistivity of YBCO is very low, not reaching 1. Therefore, the quenching characteristics and current distribution characteristics of this condition are the same as those in partial resistive state. According to Figure 13b, when *t*_2_ ≤ *t*, the temperature of YBCO layer exceeds the critical temperature *T_C_*. Its maximum temperature is 92.3 K, which results in the quenching resistivity of YBCO transformed into constant resistivity in Equation (9). At this time, the ratio of real-time resistivity to normal resistivity of YBCO is 1, which indicates that YBCO layer transitions from partial resistive state to normal state. Therefore, current mainly flows through other layers except YBCO layer. During this period, the characteristics of current distribution is: *I_Cu_* > *I_Ag_* > *I_St_* > *I_Sub_* > *I_YBCO_*. The superconducting tape further presents a composite conventional metal resistivity. However, with the decline of AC overcurrent, the superconducting tape gradually reaches thermal equilibrium. The temperature fluctuation near 90 K causes the quenching degree of YBCO layer to be in a critical state, fluctuating between partial resistive state and normal state.

**Figure 14:**
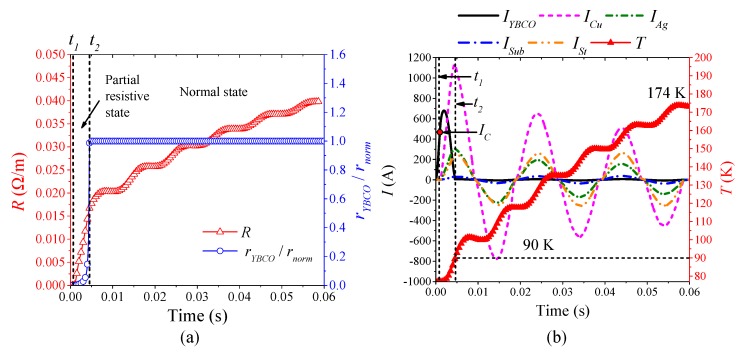
The simulation analysis with AC 1780 A overcurrent: (**a**) the quenching resistance characteristics; and (**b**) the thermal characteristics and current distribution characteristics.

Figure 14 is the simulation results of the superconducting tape quenching under AC 1780 A, which represent the characteristics of continuous incremental curve type quenching resistance in normal state. In this condition, the duration of partial resistance state is very short. Current rapidly exceeds the critical current of YBCO layer. Temperature correspondingly exceeds critical temperature 90 K within first current half wave, thereby resulting in YBCO layer transformed into normal state within about 5 ms, as shown in Figure 14. With temperature continuously rising, the superconducting tape presents corresponding incremental curve type quenching resistance. The maximum temperature of YBCO layer is 174 K. During most of quenching process, the current distribution in superconducting tape is mainly: *I_Cu_* > *I_Ag_* > *I_St_* > *I_Sub_* > *I_YBCO_*.

#### 4.3.2. Resistance, Thermal and Current Distribution Characteristics under DC Overcurrent.

The two typical quenching resistance waveforms of superconducting are found under DC overcurrent in Section 3.3 including half-wave type and incremental curve type. Therefore, the simulation results with DC 910 A and DC 2020 A were selected to analyze the transient characteristics of superconducting tape under DC overcurrent to obtain the corresponding common laws.

**Figure 15:**
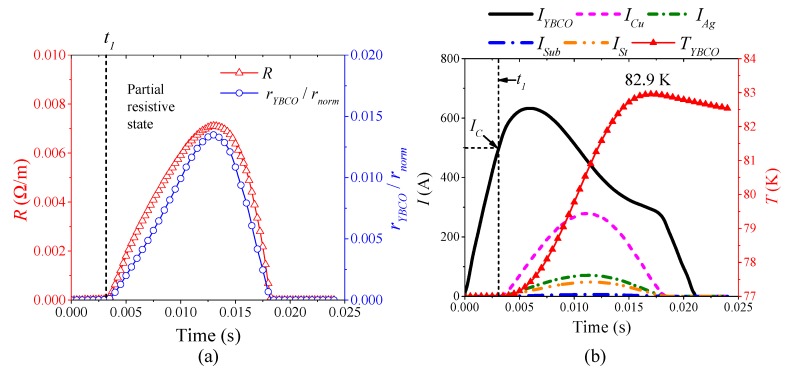
The simulation analysis with DC 910 A overcurrent: (**a**) the quenching resistance characteristics; and (**b**) the thermal characteristics and current distribution characteristics.

**Figure 16:**
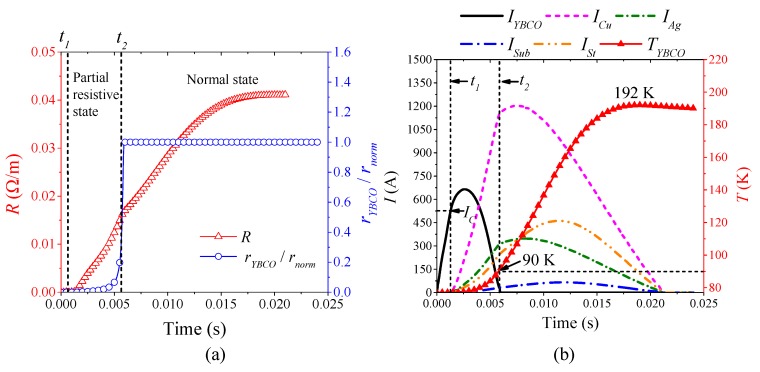
The simulation analysis with DC 2020 A overcurrent: (**a**) the quenching resistance characteristics; and (**b**) the thermal characteristics and current distribution characteristics.

Figure 15 is the simulation results of the superconducting tape quenching under DC 910 A, which represent the characteristics of half-wave type quenching resistance in DC partial resistive state, whose quenching characteristics are same as those of AC partial resistive state. When 0 ≤ *t* < *t*_1_, the superconducting tape is in superconducting state with 0 Ω. Therefore, DC overcurrent only flows through YBCO layer. When *t*_1_ ≤ *t*, DC overcurrent exceeds the critical current of YBCO layer, but the temperature is still below the critical temperature 90 K. During the whole quenching process, the maximum temperature of YBCO layer is 82.9 K. Therefore, the quenching resistivity of YBCO layers is far below normal resistivity, as shown in Figure 16a, resulting in the superconducting tape recovering quickly with DC overcurrent attenuation, thereby forming half-wave type quenching resistance. The current distribution characteristic of superconducting tape is: *I_YBCO_* > *I_Cu_* > *I_Ag_* > *I_St_* > *I_Sub_* in Figure 15b).

Figure 16 is the simulation results of the superconducting tape quenching under DC 2020 A, which represent the characteristics of incremental curve type quenching resistance in DC normal state. The quenching characteristics are the same as those of AC normal state. Because the temperature of YBCO layer exceeds the critical temperature and increases continuously, the resistivity of YBCO layer quickly transits from partial resistive state to normal state, as shown in Figure 16a. Therefore, current only flows through other layers except YBCO layer. The equivalent quenching resistance of the superconducting tape is composite resistance of metal materials depending on the temperature, which presents incremental curve type with temperature rising. Although DC overcurrent starts to decay after the first crest, the temperature is maintained at 192 K. Therefore, the superconducting tape remains at normal resistivity state, whose recovery is very slow. In this condition, current distribution characteristics is: *I_Cu_* > *I_Ag_* > *I_St_* > *I_Sub_* > *I_YBCO_* (Figure 16b).

### 4.4. Consistency Analysis of R-Q Curve

**Figure 17:**
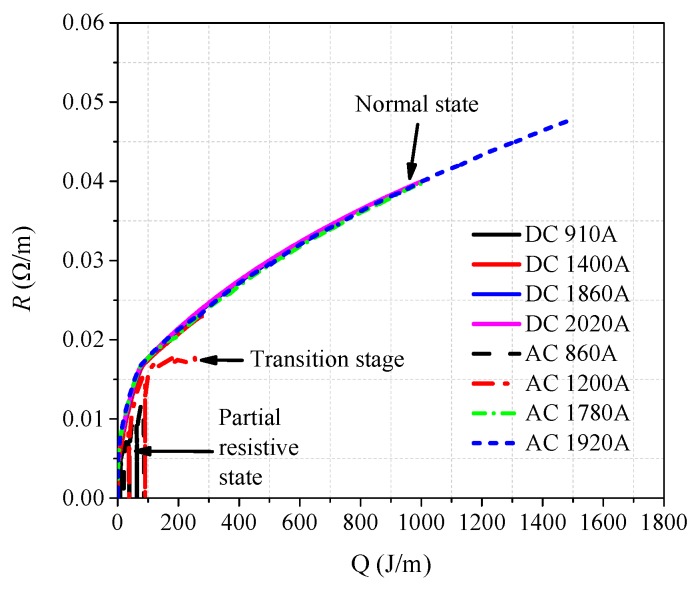
The simulation results of *R-Q* curves.

According to the simulation results shown in Figure 11, Equation (2) is adopted to plot the *R-Q* curves shown in Figure 17, which present the same phenomenon as the experimental results. When superconducting tape is in normal state in a cryogenic medium, the variation tendency of *R-Q* curves under different AC and DC overcurrent is consistent, while the partial resistive state and transition phenomena do not appear.

**Figure 18:**
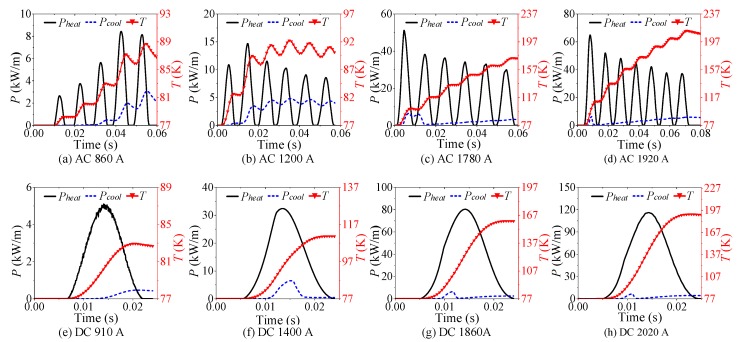
The simulation analysis of heat transfer process of 1 m superconducting tape under AC and DC overcurrent.

To explain the phenomena and analyze the consistency in *R-Q* curves in normal state, the heat transfer process of each simulation result is plotted in Figure 18, including heat power *P_heat_* generated by superconducting tape, cooling power *P_cool_* of liquid nitrogen on superconducting tape surface and temperature *T* of superconducting tape. In the consideration of the heat exchange process between superconducting tape and liquid nitrogen, accumulated joule heat *Q*_1_ can be calculated by Equation (14).

(14) $$Q_{1}\left(t_{2}\right)=\int_{t_{1}}^{t_{2}}\left(P_{heat}-P_{cool}\right)dt$$

(15) $$Q_{1}=cm\Delta T$$

where *c* is specific heat capacity, *m* is mass, and Δ*T* is equal to *T-T_ref_*. According to Equation (15), accumulated joule heat *Q*_1_ directly causes the temperature *T* to rise, which can further aggravate quenching.

When superconducting tape is in partial resistive state or transition, the temperature of superconducting tape is below 100 K. At this time, liquid nitrogen is in free convection, nuclear boiling or transition boiling. During this time, the cooling power of liquid nitrogen is relatively high, which greatly weakens the quench of superconducting tape. Therefore, the effect of *P_cool_* cannot be ignored, as shown in Figure 18a,b,e. In addition, compared with the heating power of superconducting tape, the cooling power of liquid nitrogen shows obvious hysteresis phenomenon. Therefore, heating power increases and decreases rapidly with the change of overcurrent, resulting in temperature rising first and then dropping. During partial resistive state and transition, *R-Q* curves without considering *P_cool_* cannot correctly reflect the changing rule of quenching resistance, which further validates and explains that the *R-Q* curves are not consistent at these quenching stages in the experimental results in Figure 8b.

When superconducting tape is in normal state, the surface temperature of the superconducting tape is very high, causing the surrounding liquid nitrogen to be in film boiling. Because the cooling power of liquid nitrogen is much less than the heating power of superconducting tape (*P_heat_* >> *P_cool_*), the thermal equilibrium is quickly broken by a huge thermal shock. Hence, superconducting tape is approximately in an adiabatic environment, whose heat exchange with liquid nitrogen can be ignored, as shown in Figure 18c,d,f–h. At this time, the superconducting tape is in thermal runaway; quenching resistance increases rapidly with temperature rising. *P_cool_* can be omitted, thus the amount of joule heat *Q* generated by superconducting tape can be approximately equivalent to accumulated joule heat *Q*_1_.

**Figure 19:**
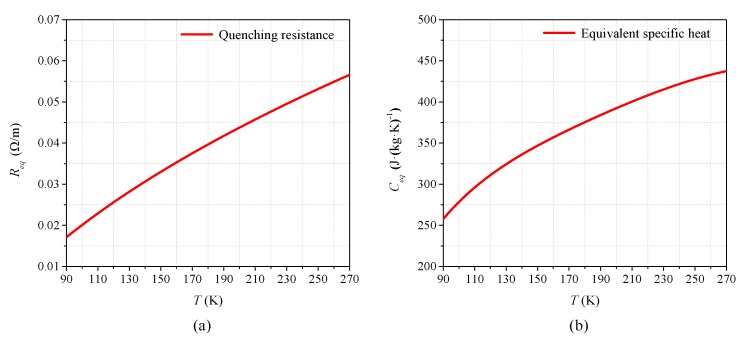
Equivalent material properties: (**a**) the equivalent quenching resistance curve; and (**b**) the equivalent specific heat capacity.

In the complete quenching process of superconducting tape, due to *T* > *T_c_*, the quenching resistivity of YBCO layer is normal resistivity *r_norm_*. The resistivity of other materials have fixed curves varying with temperature *T,* as shown in the Appendix. The equivalent resistivity of the superconducting tape can be calculated by Equation (16).

(16) $$r_{eq}=1/\left(\sum_{i=1}^{n}\frac{f_{i}}{r_{i}}\right)$$

(17) $$R_{eq}=\frac{r_{eq}\cdot l}{S}$$

where *r_i_* is the resistivity of each material layer. *f_i_* is the volume percentage of each material in the superconducting tape. *l* is the length of superconducting tape and *S* is the cross-section area. According to Equations (16) and (17), the equivalent resistance of 1 m superconducting tape varying with temperature is a fixed curve, as shown in Figure 19a.

The specific heat capacity *c* of each material has a fixed curve varying with temperature *T*, as shown in the Appendix. The equivalent specific heat capacity *c_eq_* of the superconducting tape can be calculated by Equations (18), as shown in Figure 19b.

(18) $$\left\{ \begin{array}{l} c_{v}=1/\left(\sum_{i=1}^{n}f_{i}/\left(\gamma_{i}\cdot c_{i}\right)\right)\\ c_{p}=\sum_{i=1}^{n}f_{i}\cdot \gamma_{i}\cdot c_{i}\\ c_{eq}=\left(c_{v}+c_{p}\right)/2 \end{array}\right.$$

where *c_v_* is the equivalent specific heat capacity in the vertical direction of the superconducting tape surface, *c_p_* is the equivalent specific heat capacity in the parallel direction of the superconducting tape surface, *c_i_* is the specific heat capacity of each material, and *γ_i_* is the mass density ratio of each material layer to the whole superconducting tape.

**Figure 20:**
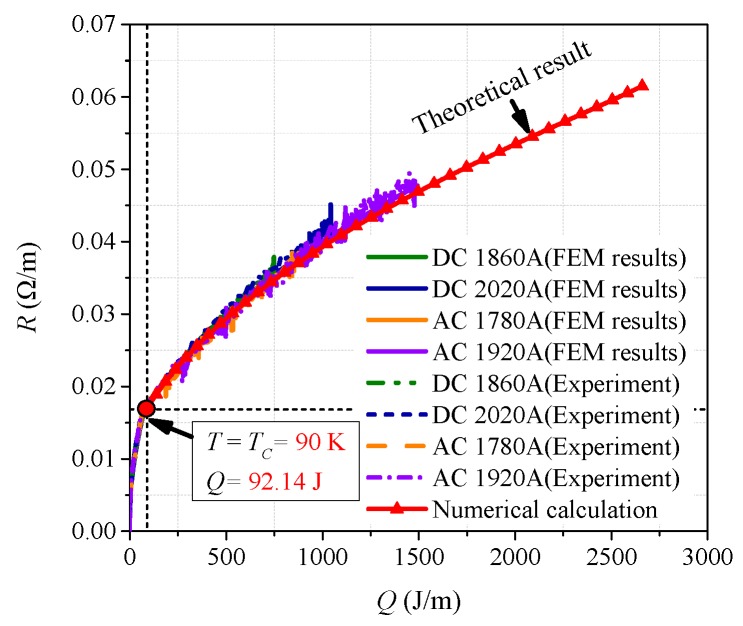
The comparison diagram of *R-Q* curves from different results.

With the superconducting tape quenching to normal state, the temperature of superconducting tape is mainly affected by the amount of joule heat generated with time. Therefore, during this condition, the *R-Q* curves of superconducting tape under different AC and DC overcurrent are consistent, which can be used to reflect the change law of quenching resistance. According to Equations (14)–(18), the theoretical *R-Q* curve in normal state can be calculated by numerical calculation, which is compared with experiment results and FEM results in Figure 20.

The comparison in Figure 20 obviously indicates that, when *T* > *T_C_*, the *R-Q* curves of superconducting tape are basically consistent with the theoretical result. In normal state, critical temperature *T_C_* is the turning point of quenching resistance variation. The accumulated joule heat required is about 92.14 J. Furthermore, it also confirms the correctness of the theoretical analysis of *R-Q* curves consistency mentioned above. When the superconducting tape enters normal state, it is approximately in an adiabatic environment, and quenching resistance will vary with the accumulated joule heat along a fixed *R-Q* curve, which is consistent under AC and DC overcurrent.

### 4.5. The Summary of Quenching Characteristics under AC and DC Overcurrent

**Figure 21:**
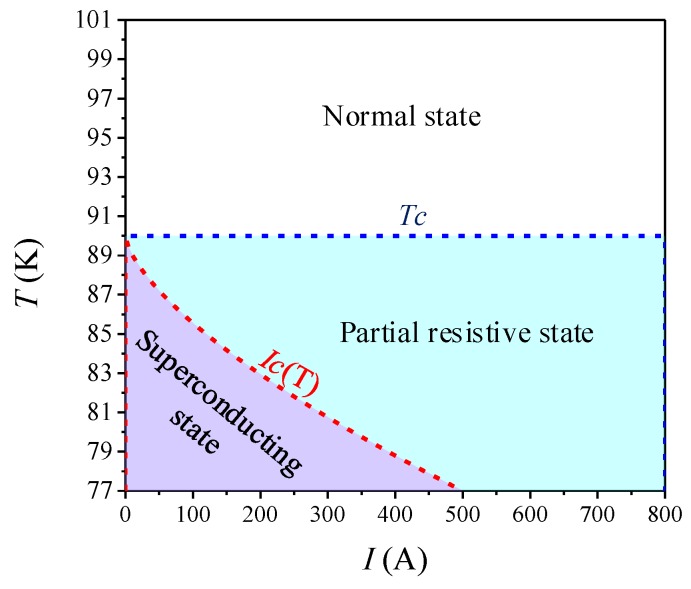
Different stage distribution of superconducting tape immersed in liquid nitrogen.

Based on the above simulation and experimental analysis, the superconducting tape is divided into three stages: superconducting state, partial resistive state and normal state. Their classified method is shown in Figure 21 and corresponding characteristics are listed in Table 7. Critical current *I_C_(T)* is the criterion of whether or not to quench. Critical temperature *T_C_* is the criterion of quenching degree. The consistency of *R-Q* curves is satisfied in normal state.

## 5. The Equivalent Estimation of Quenching Resistance under AC and DC Overcurrent

### 5.1. The Basic Principle of the New Method

Combined with experiment and FEM simulation, a general rule is further verified. When superconducting tape quenches to normal state, immersed in liquid nitrogen environment, the superconducting tape has a correspondence relationship between quenching resistance and the accumulated joule heat. The *R-Q* curve is consistent under different AC and DC overcurrent. Therefore, a new calculation method of quenching resistance is proposed to estimate and predict variation of quenching resistance when superconducting tape is in normal state. The joule heat adopted in the new method can effectively evaluate the quenching resistance of superconducting tape and ignore the difference of current waveforms.

**Figure 22:**
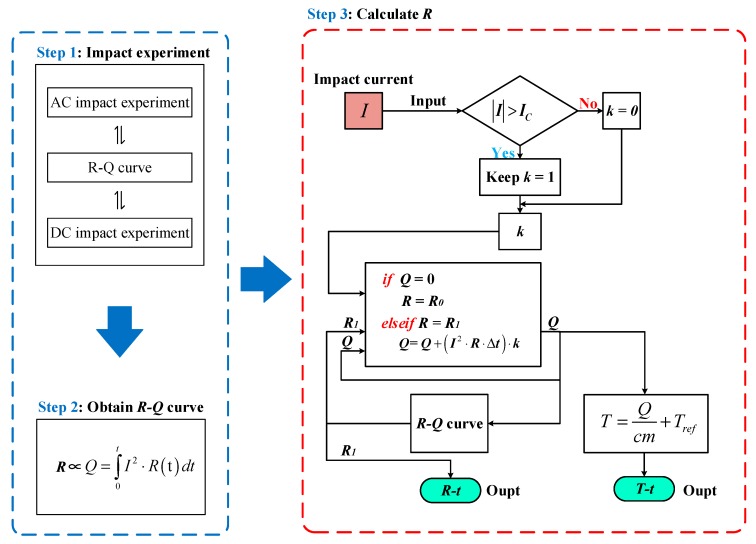
The calculation process of *R-Q* curve method.

The method is defined as “*R-Q* curve method”, whose calculation process is shown in Figure 22. In the calculation process, test current is used as input current. *I_C_* is quenching criterion. *R*_0_ is set as initial quenching resistance for initial energy accumulation. Δ*t* is the time step of calculation. When superconducting tape starts to quench, the accumulated joule heat *Q* in each time step is calculated by discrete calculation, whose corresponding quenching resistance *R* can be obtained by interpolation calculation based on *R-Q* curve. In addition, under overcurrent shock, the effect of liquid nitrogen on superconducting tape can be approximately ignored. Therefore, the temperature variation of superconducting tape can be calculated by Equation (19).

(19) $$T=\frac{Q}{cm}+T_{ref}$$

### 5.2. The Validation of R-Q Curve Method

**Figure 23:**
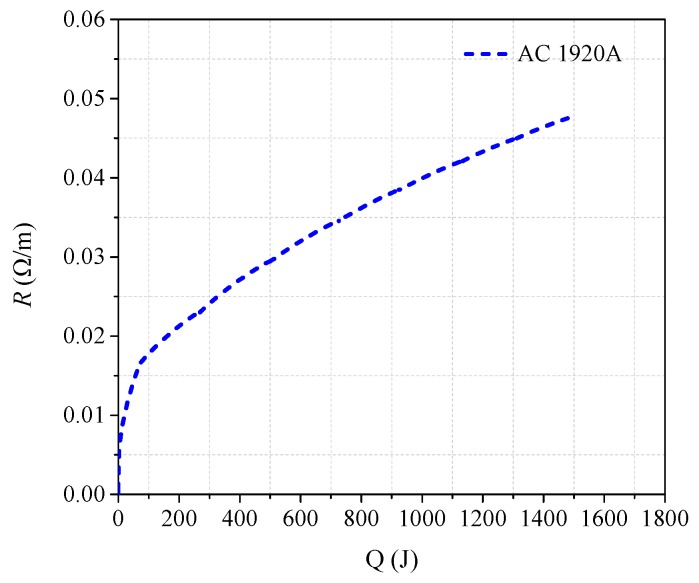
*R-Q* curve required in simulation calculation.

According to Section 3 and Section 4, under AC and DC overcurrent, the *R-Q* curves of superconducting tape obtained have the same trend. Therefore, the experimental data with the most extensive coverage are adopted to be converted into the *R-Q* curve of per meter superconducting tape, as shown in Figure 23, whose data are from AC 1920 A overcurrent test. The simulation parameters setting are listed in Table 8.

**Figure 24:**
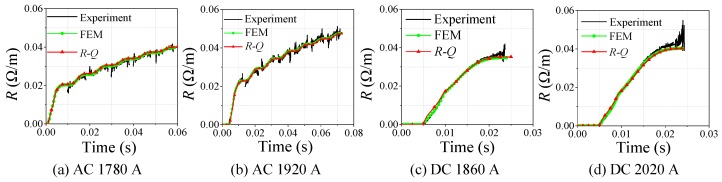
The quenching resistance comparison of FEM method, *R-Q* curve method and experiment.

**Figure 25:**
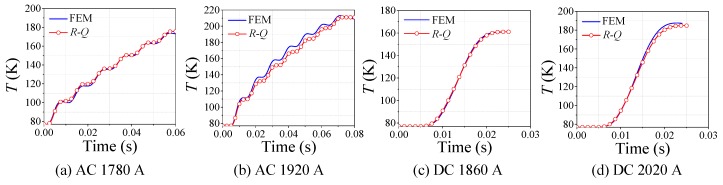
The temperature comparison of FEM and *R-Q* curve method.

According to Figure 24, with comparison between simulations and experiments, the effectiveness of *R-Q* curve method is well verified, which can be used to simulate the variation process of quenching resistance of small-scale superconducting tape in normal state under AC and DC overcurrent. In addition, the *R-Q* curve in the simulation comes from the AC experimental data, but the model of *R-Q* curve method still has good simulation accuracy under DC overcurrent. It also indicates that the quenching resistance of AC and DC overcurrent test can be equivalent by *R-Q* curve in normal state. Meanwhile *R-Q* curve method can calculate temperature change in the quenching process, whose results are basically consistent with the results of FEM but computing speed is faster, as shown in Figure 25.

Obviously, in terms of estimating quenching resistance of superconducting tape, *R-Q* curve method has a good calculation accuracy in normal state. However, compared with FEM, because of *R-Q* curve method using interpolation calculation while FEM adopting the coupling calculation of multiple physical fields, the model structure of *R-Q* curve method is simpler and faster, as shown in Table 9. However, it is undeniable that the application range of *R-Q* curve method is limited.

Having good simulation precision and calculation speed, the *R-Q* method can be applied to evaluate and predict the quenching resistance of large-scale non-inductive superconducting coil in R-SFCL based on the experimental data of small-scale superconducting tape. The prediction results of the model is compared with the experimental results of the coil to verify this estimation method.

**Figure 26:**
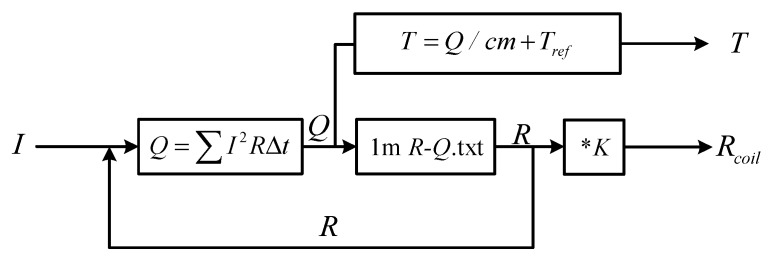
The model of *R-Q* curve method improved in PSCAD.

To calculate the quenching resistance of superconducting tape with any length, the model of *R-Q* curve method is improved in PSCAD, as shown in Figure 26, whose *R-Q* curve data comes from the experimental results of 10 cm superconducting tape converted to the *R-Q* curve of 1 m superconducting tape applied in the model. The coefficient *K* is the length of superconducting tape, which can be set according to the length of experimental sample including the length of superconducting tape in non-inductive superconducting coil.

**Figure 27:**
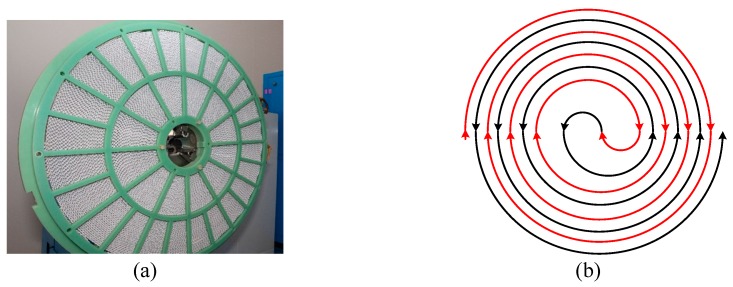
The schematic diagram of non-inductive superconducting coil: (**a**) physical drawing; and (**b**) structure drawing.

**Figure 28:**
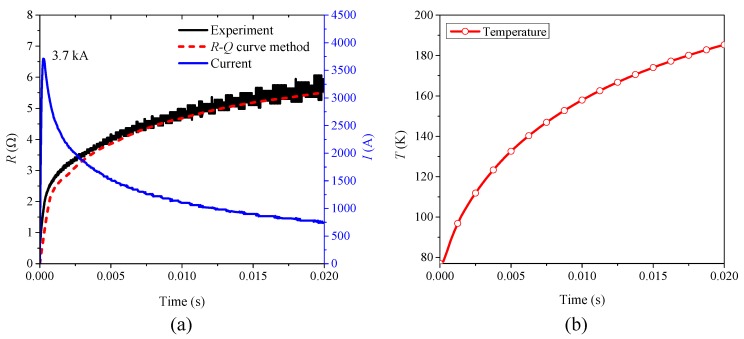
Comparison between the result of *R-Q* curve method and experimental result of 136 m non-inductive superconducting coil: (**a**) quenching resistance; and (**b**) temperature estimation.

The non-inductive superconducting coil is shown in Figure 27, which adopts single 12 mm width superconducting tape winding. Its diameter is 1200 mm and its length of superconducting tape is 136 m. Because the superconducting coil adopts non-inductive design, the effect of magnetic field on superconducting tape can be neglected. According to Figure 28, the peak value of DC overcurrent is 3.7 kA, which results in superconducting coil rapidly entering in complete quenching stage. The inductance of superconducting non-inductive coil can be ignored, but its resistance needs to be considered, where resistance and accumulated joule heat are proportional to the length of superconducting tape. Without considering the influence of coil structure, the quenching resistance of 136 m superconducting tape calculated by *R-Q* curve method is basically consistent with the experimental results of actual superconducting coil. It indicates that the non-inductive coil structure can effectively promote the uniform quench of the superconducting coil.

In addition, Figure 28 clearly shows that *R-Q* curve method has good simulation accuracy and fast solving speed compared with the experimental results in the time range of tens of milliseconds. The *R-Q* method can rapidly calculate the quenching resistance and average temperature of large non-inductive superconducting coils during complete quenching, only taking a few seconds. Therefore, the method can be improved to predict the dynamic quenching resistance and temperature of superconducting coil for R-SFCL design.

## 6. Conclusions

In this paper, through the combination of AC and DC overcurrent experiment and simulation study, the quenching characteristics and common laws of YBCO superconducting tape applied in R-SFCL are summarized as follows:

(1) Under AC overcurrent, the quenching resistance of the HTS tape can be divided into three types: half-wave type, half-wave and half-incremental curve type and incremental curve type. Under the DC overcurrent, there are two quenching resistance types of the HTS tape: half-wave type and incremental curve type.

(2) The quench resistance type is closely related to the current and the temperature of the HTS tape. When the current exceeds the critical current and the temperature is lower than the critical temperature of the HTS tape, the HTS tape is in partial resistance state. When the temperature exceeds the critical temperature, the HTS tape enters normal state.

(3) In the normal state, the relationship between quenching resistance *R* and joule heat *Q* of HTS tape and coil is independent of current waveform. In essence, there is a fixed correspondence between *R* and *Q*. Therefore, *R-Q* curves are consistent under AC and DC overcurrent.

Based on these, the *R-Q* curve method can be used to estimate and predict the quench resistance and temperature change of HTS tapes in completely quenching stage. Compared with FEM, this method can ensure simulation accuracy, greatly shorten calculation time and improve simulation efficiency. It can be used for simulation modeling, prototype design and quench resistance estimation of resistive type SFCL in complex power systems. Furthermore, this method can realize the equivalent calculation of quenching resistance under AC and DC overcurrent, which can provide theoretical support for large-scale DC current limiters to be tested by AC equivalent experiment.

## Figures and Tables

**Table 1:** Material parameters of 2G HTS tape used in experiment.

Parameters	Value
Width of tape	12 mm
Thickness of stainless steel layer	80 μm
Thickness of Cu layer	3 μm
Thickness of Ag layer	1.5 μm
Thickness of YBCO layer	1 μm
Thickness of Hastelloy substrate layer	50 μm
Insulation	No
Unit resistance at room temperature	58.42 mΩ/m
Self-field critical current Ic at 77 K	500 A
Effective measurement length of Sample	10 cm

**Table 2:** Basic parameters of AC overcurrent test platform.

AC Test Platform		DC Test Platform	
Parameters	Value	Parameters	Value
Input voltage	220 V	Output voltage *U_C_*	1000–2200 V
Frequency	50 Hz	Capacitance *C*_0_	8 mF
Output voltage	4–6 V	Inductance *L*_0_	5 mH
Output current	0–3 kA	Resistance *R*_0_	0.2 Ω/0.5 Ω
Duration	50–80 ms	Duration	20–23 ms

**Table 3:** The parameters setting of AC and DC overcurrent tests.

Number	*I_Pmax_* (A)		*I_Pmax_*/*I_C_*		Duration (ms)	
AC/DC	AC	DC	AC	DC	AC	DC
No. 1	860	910	1.72	1.82	50	22
No. 2	1060	1260	2.12	2.52	60	23
No. 3	1200	1400	2.4	2.8	60	20
No. 4	1420	1580	2.84	3.16	70	20
No. 5	1580	1620	3.16	3.24	80	22
No. 6	1620	1860	3.24	3.72	60	22
No. 7	1780	1980	3.56	3.96	60	22
No. 8	1920	2020	3.84	4.04	75	22

**Table 4:** The summary of AC overcurrent experiments.

Resistance Type	Waveform	Results	Characteristics	State
Half-wave type	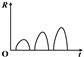	AC No. 1	Recovery after quenching	Partial resistive
Half-wave and half-incremental curve type	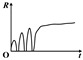	AC No. 2 AC No. 3	Initial stage: Recovery after quenching; Later stage: continuously quenching	Transition: Partial resistive state transitions to normal state
Incremental curve type	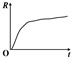	AC No. 4 AC No. 5 AC No. 6 AC No. 7 AC No. 8	Continuously quenching to normal state	Normal

**Table 5:** The summary of DC overcurrent experiments.

Resistance Type	Waveform	Results	Characteristics	State
Half-wave type	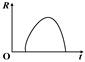	DC No. 1	Recovery after quenching	Partial resistive
Incremental curve type	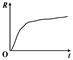	DC No. 2 DC No. 3 DC No. 4 DC No. 5 DC No. 6 DC No. 7 DC No. 8	Continuously quenching to normal state	Normal

**Table 6:** Simulation parameters of YBCO resistivity.

Parameters	Value	Parameters	Value
*r*_0_	1 × 10^−14^ Ω·cm	*n*_1_	2.8
*r_norm_*	2.5 μΩ·cm	*n*_2_	22
*E*_0_	0.5 V/cm	*k*	1.92
*T_c_*	90 K	*α*	1.5
*J_c_*_0_	1.9 × 10^10^ A/m^2^	*T_ref_*	77 K

**Table 7:** The common quenching characteristics of the superconducting tape.

State		Boundary	Current Distribution Characteristics	YBCO Resistivity	*R-Q* Consistency
Superconducting state		*I* < *I_C_* (*T*)	Only flow through superconducting layer	0	/
Quenching	Partial resistive state:	*I* > *I_C_* (*T*) *T* < *T_C_*	Initial stage: *I_YBCO_* > *I_Cu_* > *I_Ag_* > *I_St_* > *I_Sub_* Late stage: *I_Cu_* > *I_YBCO_* > *I_Ag_* > *I_St_* > *I_Sub_*	$\frac{\left(r_{1}+r_{2}+r_{0}\right)r_{norm}}{r_{0}+r_{1}+r_{2}+r_{norm}}$	No
	Normal state	*T_C_ < T*	*I_Cu_* > *I_Ag_* > *I_St_* > *I_Sub_* > *I_YBCO_*	$r_{norm}$	Yes

**Table 8:** Simulation parameters setting of *R-Q* curve method.

Parameters	Value
Critical current, *I_C_*	500 A
Initial quenching resistance, *R*_0_	0.001 Ω
Time step, Δ*t*	5 × 10^−5^ s
Length of superconducting tape	1 m
Overcurrent	AC 1780 A, AC 1920 A, DC 1860 A, DC 2020 A,
Duration	60 ms, 80 ms, 25 ms, 25 ms

**Table 9:** The computation time of *R-Q* curve method and FEM.

Overcurrent	Software	Method	Computation Time
AC 1780A, 60 ms	PSCAD	*R-Q*	<3 s
	Comsol	FEM	3.78 h
AC 1920A, 75 ms	PSCAD	*R-Q*	< 3 s
	Comsol	FEM	3.85 h
DC 1860A, 22 ms	PSCAD	*R-Q*	<3 s
	Comsol	FEM	1.57 h
DC 2020A, 22 ms	PSCAD	*R-Q*	<3 s
	Comsol	FEM	1.58 h

**Figure A1:**
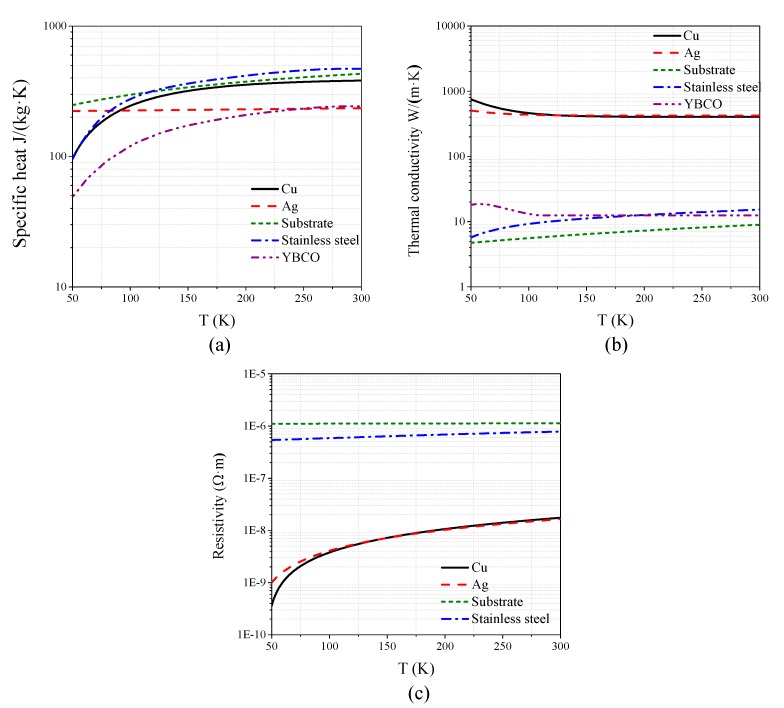
Material properties: (**a**) specific heat capacity of each layer of material; (**b**) thermal conductivity of each layer of material; and (**c**) resistivity of each layer of material.

## Nomenclature

BSCCO	Bismuth Strontium Calcium Copper Oxide
YBCO	Yttrium Barium Copper Oxide
1G	First Generation
2G	Second Generation
3D	Three Dimensional
HTS	High Temperature Superconducting
R-SFCL	Resistance type Superconducting Fault Current Limiter
DC	Direct Current
AC	Alternating Current
VSC	Voltage Source Converter
MMC	Modular Multilevel Converter
HVDC	High Voltage Direct Current
FEM	Finite element method
PDE	Partial Differential Equation
RLC	Resistance (R), Inductance (L) and Capacitance (C)

## Symbols

*U*	Voltage (V)	*Q*	Joule heat (J)
*I*	Current (A)	*P*	Power (W)
*R*	Resistance (Ω)	*E*	Electric field intensity (V/m)
*L*	Inductance (H)	*J*	Current density (A/m^2^)
*C*	Capacitance (F)	*r*	Resistivity (Ω·m)
*t*	Time (s)	*q_s_*	Volume power density (W/m)
*m*	Mass (kg)	*k*	Heat transfer coefficient (W/(m^2^·K))
*ρ*	Mass density (kg/m^3^)	*μ*	Permeability (H/m)
*c*	Specific heat capacity (J/(kg·K))	*H*	Magnetic field intensity (A/m)
*q*	Conduction heat flux (W/m^2^)	*B*	Magnetic induction intensity (T)
*T*	Temperature (K)		

## Appendix A

The following are the material properties required in the simulation, which mainly come from [46,49,50]. After collection and collation, the properties of materials are shown in Figure A1 and Table A1.

**Table A1:** Mass density of each material in superconducting tape.

Mass Density (kg/m^3^)			
Cu	8940	YBCO	5900
Ag	10500	Stainless steel	7930
Substrate	8910		

## References

[1] Malozemoff A.P. (2012). Second-Generation High-Temperature Superconductor Wires for the Electric Power Grid. Annu. Rev. Mater. Res..

[2] Rogalla H., Kes P.H. (2011). 100 Years of Superconductivity.

[3] Ronald M., Alexis P., David C. (2004). Superconducting Materials for Large Scale Applications. IEEE Trans. Appl. Supercond..

[4] Malozemoff A.P., Verebelyi D.T. (2003). HTS Wire: Status and prospects. Phys. C. Superconduct..

[5] Chen Y., Bian W., Huang W. (2016). High critical current density of YBa_2_Cu_3_O_7-x_ superconducting films prepared through a DUV-assisted solution deposition process. Sci. Rep..

[6] Schoop U., Rupich M.W., Thieme C. (2005). Second generation HTS wire based on RABiTS substrates and MOD YBCO. IEEE Trans. Appl. Supercond..

[7] Rupich M.W., Schoop U., Verebelyi D.T. (2007). The Development of Second Generation HTS Wire at American Superconductor. IEEE Trans. Appl. Supercond..

[8] Pascal P.T., Badel A., Auran G. (2017). Superconducting Fault Current Limiter for Ship Grid Simulation and Demonstration. IEEE Trans. Appl. Supercond..

[9] Yang K., Yang Y., Junaid M. (2018). Direct-Current Vacuum Circuit Breaker with Superconducting Fault-Current Limiter. IEEE Trans. Appl. Supercond..

[10] Chen L., Zhang X., Qin Y. (2019). Application and Design of a Resistive-Type Superconducting Fault Current Limiter for Efficient Protection of a DC Microgrid. IEEE Trans. Appl. Supercond..

[11] He H., Chen L., Yin T. (2016). Application of a SFCL for Fault Ride-Through Capability Enhancement of DG in a Microgrid System and Relay Protection Coordination. IEEE Trans. Appl. Supercond..

[12] Li B., He J. (2016). Studies on the Application of R-SFCL in the VSC-Based DC Distribution System. IEEE Trans. Appl. Supercond..

[13] Chen L., Chen H., Shu Z. (2016). Comparison of Inductive and Resistive SFCL to Robustness Improvement of a VSC-HVDC System with Wind Plants against DC Fault. IEEE Trans. Appl. Supercond..

[14] Xiao L., Dai S., Lin L. (2013). HTS Power Technology for Future DC Power Grid. IEEE Trans. Appl. Supercond..

[15] Zhou Y., Song Q., Guo F. (2005). Quench developing process of HTS tapes under sinusoidal over-currents. IEEE Trans. Appl. Supercond..

[16] Baldan C.A., Lamas J.S., Shigue C.Y. (2011). Test of a Modular Fault Current Limiter for 220 V Line Using YBCO Coated Conductor Tapes with Shunt Protection. IEEE Trans. Appl. Supercond..

[17] Liu X., Wen J., Zeng W. (2015). Quenching Characteristics of Different Types of Superconducting Fault Current Limiting Modules. IEEE Trans. Appl. Supercond..

[18] Zhang Z., Yang J., Qiu Q. (2017). Research on resistance characteristics of YBCO tape under short-time DC large current impact. Cryogenics.

[19] Xiang B., Zhang L., Tan Y. DC current withstanding characteristics of superconductor. Proceedings of the 3rd International Conference on Electric Power Equipment—Switching Technology.

[20] Jiang Z., Wang Y., Dai S. (2018). Influence of Insulation on Quench and Recovery of YBCO Tape under DC Impact. IEEE Trans. Appl. Supercond..

[21] Xiang B., Junaid M., Gao L. (2018). Influencing Factors on Quench and Recovery of YBCO Tapes for DC Superconducting Fault Current Limiter. IEEE Trans. Appl. Supercond..

[22] Xiang B., Junaid M., Gao L. (2018). Effects of Short Circuit Currents on Quench and Recovery Properties of YBCO Tapes for DC SFCL. IEEE Trans. Appl. Supercond..

[23] Badel A., Antognazza L., Decroux M. (2013). Hybrid Model of Quench Propagation in Coated Conductors Applied to Fault Current Limiter Design. IEEE Trans. Appl. Supercond..

[24] Tong Y., Guan M., Wang X. (2017). Theoretical estimation of quench occurrence and propagation based on generalized thermoelasticity for LTS/HTS tapes triggered by a spot heater. Supercond. Sci. Technol..

[25] Colangelo D., Dutoit B. (2014). Analysis of the influence of the normal zone propagation velocity on the design of resistive fault current limiters. Supercond. Sci. Technol..

[26] Núñez-Chico A.B., Martínez E., Angurel L.A., Navarro R. (2016). Enhanced quench propagation in 2G-HTS coils co-wound with stainless steel or anodised aluminium tapes. Supercond. Sci. Technol..

[27] Hong Z., Sheng J., Yao L., Gu J., Jin Z. (2012). The Structure, Performance and Recovery Time of a 10 kV Resistive Type Superconducting Fault Current Limiter. IEEE Trans. Appl. Supercond..

[28] Ge H., Yang K., Junaid M. A quenching recovery time test method for resistive type superconducting fault current limiters used in DC circuit. Proceedings of the 4th International Conference on Electric Power Equipment—Switching Technology.

[29] Yang D.G., Song J.B., Choi Y.H. (2011). Quench and Recovery Characteristics of the Zr-Doped (Gd,Y) BCO Coated Conductor Pancake Coils Insulated With Copper and Kapton Tapes. IEEE Trans. Appl. Supercond..

[30] Lim S.H., Lim S.T. (2018). Current Limiting and Recovery Characteristics of a Trigger-Type SFCL Using Double Quench. IEEE Trans. Appl. Supercond..

[31] Schwarz M., Schacherer C., Weiss K.P. (2008). Thermodynamic behaviour of a coated conductor for currents above Ic. Supercond. Sci. Technol..

[32] Bagrets N., Otten S., Weiss K.P. (2015). Thermal and mechanical properties of advanced impregnation materials for HTS cables and coils. IOP Conf. Ser. Mater. Sci. Eng..

[33] Bae J.H., Eom B.Y., Sim K.D. (2013). Minimum Quench Energy Characteristic of YBCO Coated Conductor with Different Stabilizer Thickness. IEEE Trans. Appl. Supercond..

[34] Falorio I., Young E.A., Yang Y. (2015). Quench Characteristic and Minimum Quench Energy of 2G YBCO Tapes. IEEE Trans. Appl. Supercond..

[35] Du H.I., Kim Y.J., Lee D.H. (2010). Study on Maximum Operating Condition of Resistive Type SFCL Using YBCO Coated Conductor. IEEE Trans. Appl. Supercond..

[36] Levin G.A., Jones W.A., Novak K.A. (2011). The effects of superconductor-stabilizer interfacial resistance on quenching of a pancake coil made out of coated conductor. Supercond. Sci. Technol..

[37] Kwon N.Y., Kim H.S., Kim K.L. (2010). The Effects of a Stabilizer Thickness of the YBCO Coated Conductor (CC) on the Quench/Recovery Characteristics. IEEE Trans. Appl. Supercond..

[38] Du H. (2013). Evaluation on Resistance Tendency and Recovery Characteristics of 2G Wire with Insulation Layer. IEEE Trans. Appl. Supercond..

[39] The Shanghai Superconductor Website 2019. http://www.shsctec.com/en/index.

[40] Yang J., Fletcher J., O’Reilly J. (2012). Short-Circuit and Ground Fault Analyses and Location in VSC-Based DC Network Cables. IEEE Trans. Ind. Electron..

[41] Li C., Zhao C., Xu J. (2017). A Pole-to-Pole Short-Circuit Fault Current Calculation Method for DC Grids. IEEE Trans. Power Syst..

[42] Hong Z., Campbell A.M., Coombs T.A. (2006). Numerical solution of critical state in superconductivity by finite element software. Supercond. Sci. Technol..

[43] Duron J., Grilli F., Antognazza L. (2007). Finite-element modelling of YBCO fault current limiter with temperature dependent parameters. Supercond. Sci. Technol..

[44] Stavrev S., Grilli F., Dutoit B. (2002). Comparison of numerical methods for modeling of superconductors. IEEE Trans. Magn..

[45] Curras S.R., Vina J., Ruibal M. (2002). Normal-state resistivity versus critical current in YBa_2_Cu_3_O_7−δ_ thin films at high current densities. Phy. C. Superconduct..

[46] Roy F., Dutoit B., Grilli F. (2008). Magneto-Thermal Modeling of Second-Generation HTS for Resistive Fault Current Limiter Design Purposes. IEEE Trans. Appl. Supercond..

[47] Liang F., Yuan W., Baldan C. (2015). Modeling and Experiment of the Current Limiting Performance of a Resistive Superconducting Fault Current Limiter in the Experimental System. J. Supercond. Novel Magn..

[48] Frost W., Harper W.L. (1975). Heat Transfer at Low Temperatures.

[49] De Sousa W., Näckel O., Noe M. (2014). Transient Simulations of an Air-Coil SFCL. IEEE Trans. Appl. Supercond..

[50] Kalsi S.S. (2011). Applications of High Temperature Superconductors to Electric Power Equipment.

